# Supplementary material for: A Likelihood Approach for Real-Time Calibration of Stochastic Compartmental Epidemic Models
Source: PLoS Comput Biol. 2017 Jan 17;13(1):e1005257. doi: 10.1371/journal.pcbi.1005257 (PMC5240920; doi:10.1371/journal.pcbi.1005257)
Supplement: S1 File — (TAR.GZ) [file pcbi.1005257.s014.tar.gz › HSPH_Online-SI-Revision/IPoi09/n10-extreme/n10-extreme_BENCH_table0.pdf]

1	R0	{2.68692, 12.5884}	{2.99939, 13.7076}	{{2.99939, 13.7076}, {2.99939, 13.7076}}	{11.6293, 8.89072}	inf	2477.	1954.02	{1328., 2541.}	24.5725	0.131263	newly	1264.	877.242	{587., 1294.}	32.7672	0.183453	Reff	0.771415	0.497362	{0.309537, 0.703057}	51.0605	ar	9089.	9410.45	{9366., 9453.}	3.53671
2	R0	{1.79802, 12.5412}	{2.85956, 19.8999}	{{2.85956, 19.8999}, {2.85956, 19.8999}}	{59.0394, 58.6762}	inf	1208.	2393.75	{1971., 2780.}	102.9	0.311474	newly	678.	814.59	{620., 984.}	27.6077	0.114369	Reff	1.00509	0.910939	{0.482694, 1.49298}	41.4691	ar	7412.	9302.69	{9253., 9345.}	25.5085
3	R0	{2.6174, 10.1105}	{2.93906, 10.3908}	{{2.93906, 10.3908}, {2.93906, 10.3908}}	{12.2893, 2.77237}	inf	2244.	2121.07	{1558., 2758.}	18.0949	0.0848486	newly	1325.	1230.41	{829., 1761.}	21.58	0.102306	Reff	0.662726	0.575203	{0.350336, 1.01015}	40.3478	ar	9109.	9363.79	{9316., 9404.}	2.79709
4	R0	{2.27532, 14.3149}	{2.93488, 18.8445}	{{2.93488, 18.8445}, {2.93488, 18.8445}}	{28.9876, 31.6426}	inf	1860.	2388.89	{1786., 2868.}	37.0386	0.143087	newly	862.	848.18	{593., 1039.}	16.2128	0.0766059	Reff	0.820025	0.889245	{0.437297, 1.96079}	46.4381	ar	8478.	9360.51	{9324., 9401.}	10.4094
5	R0	{2.96649, 18.0674}	{2.96307, 14.3219}	{{2.96307, 14.3219}, {2.96307, 14.3219}}	{0.115288, 20.7307}	inf	2876.	1423.27	{839., 2459.}	50.6993	0.336565	newly	1043.	603.306	{334., 1060.}	47.1917	0.293992	Reff	0.663011	0.368023	{0.245046, 0.547279}	52.5332	ar	9476.	9382.58	{9331., 9427.}	0.989256
6	R0	{2.94778, 16.0002}	{2.99694, 13.6953}	{{2.99939, 13.7076}, {2.99939, 13.7076}}	{1.72898, 14.4055}	inf	2859.	2413.94	{1870., 2885.}	16.516	0.0933374	newly	1175.	1120.79	{809., 1464.}	16.9513	0.0830697	Reff	0.814766	0.695477	{0.39442, 1.10018}	41.6445	ar	9321.	9408.87	{9366., 9445.}	0.94272
7	R0	{2.17562, 11.7184}	{2.8186, 12.0293}	{{2.8186, 12.0293}, {2.8186, 12.0293}}	{29.5539, 2.65309}	inf	1758.	1981.17	{1354., 2489.}	22.2279	0.0893118	newly	1027.	1026.17	{656., 1425.}	19.6235	0.0877866	Reff	0.998827	0.609447	{0.328367, 1.0426}	52.5177	ar	8345.	9275.2	{9219., 9322.}	11.1468
8	R0	{2.7904, 17.8966}	{2.7499, 15.3124}	{{2.7499, 15.3124}, {2.7499, 15.3124}}	{1.4514, 14.4396}	inf	2748.	1804.03	{1143., 2516.}	34.3511	0.199659	newly	978.	749.952	{447., 1127.}	29.0075	0.160527	Reff	0.871163	0.548299	{0.336038, 0.841194}	46.8987	ar	9285.	9203.37	{9154., 9249.}	0.886098
9	R0	{2.51574, 16.376}	{2.86118, 15.5722}	{{2.86118, 15.5722}, {2.86118, 15.5722}}	{13.7311, 4.9084}	inf	2361.	2034.54	{1527., 2572.}	17.7085	0.0887753	newly	932.	827.918	{577., 1086.}	19.6352	0.0968885	Reff	0.84076	0.591392	{0.354786, 1.00342}	47.9137	ar	8937.	9309.66	{9255., 9350.}	4.16981
10	R0	{2.56405, 18.1787}	{2.7499, 15.3124}	{{2.7499, 15.3124}, {2.7499, 15.3124}}	{7.2483, 15.7674}	inf	2387.	1804.03	{1143., 2516.}	26.5619	0.147413	newly	979.	749.952	{447., 1127.}	29.0405	0.160799	Reff	0.996903	0.548299	{0.336038, 0.841194}	51.6334	ar	9005.	9203.37	{9154., 9249.}	2.2029
11	R0	{2.475, 17.1709}	{2.47909, 18.9738}	{{2.46477, 18.9032}, {2.46477, 18.9032}}	{0.925657, 10.4996}	inf	2191.	1854.53	{1307., 2246.}	17.2494	0.113984	newly	841.	659.033	{442., 822.}	21.9342	0.138319	Reff	0.935303	0.884759	{0.472743, 1.46876}	38.1169	ar	8870.	8900.15	{8817., 8957.}	0.693912
12	R0	{2.14974, 11.8619}	{2.8833, 17.1847}	{{2.8833, 17.1847}, {2.8833, 17.1847}}	{34.1232, 44.8731}	inf	1788.	2452.79	{1814., 2851.}	47.2109	0.18555	newly	1021.	979.754	{737., 1153.}	14.3415	0.0786584	Reff	1.0409	1.09858	{0.563108, 1.95545}	38.0311	ar	8414.	9313.56	{9263., 9361.}	10.6912
13	R0	{2.20385, 14.4767}	{2.79269, 19.984}	{{2.79269, 19.984}, {2.79269, 19.984}}	{26.7187, 38.0425}	inf	1745.	2249.73	{1560., 2735.}	38.6705	0.163794	newly	848.	752.15	{560., 943.}	16.8101	0.0991373	Reff	0.831733	0.877109	{0.461632, 1.45248}	40.0783	ar	8347.	9238.12	{9175., 9301.}	10.6759
14	R0	{2.35671, 13.8357}	{2.96307, 14.3219}	{{2.96307, 14.3219}, {2.96307, 14.3219}}	{25.7291, 3.5141}	inf	2049.	1832.69	{1179., 2572.}	22.1516	0.108111	newly	946.	802.194	{497., 1190.}	27.9377	0.140591	Reff	0.81118	0.484405	{0.284158, 0.801807}	53.4731	ar	8719.	9382.58	{9331., 9427.}	7.61074
15	R0	{1.91368, 10.1082}	{2.9701, 19.3343}	{{2.97157, 19.3547}, {2.97157, 19.3547}}	{55.2037, 91.2733}	inf	1215.	2235.02	{1563., 2887.}	85.4173	0.258989	newly	838.	762.344	{523., 1008.}	20.3236	0.0994767	Reff	0.888904	0.727049	{0.344999, 1.31248}	50.7419	ar	7486.	9387.28	{9349., 9425.}	25.3978
16	R0	{2.48727, 14.882}	{2.96947, 15.963}	{{2.96947, 15.963}, {2.96947, 15.963}}	{19.3867, 7.26381}	inf	2351.	2512.32	{1970., 2939.}	19.5071	0.0999695	newly	1082.	1054.13	{778., 1265.}	14.8858	0.0788666	Reff	0.913574	0.963468	{0.502434, 1.73536}	44.373	ar	8894.	9383.39	{9320., 9450.}	5.50244
17	R0	{2.46795, 13.1915}	{2.9884, 12.8326}	{{2.9884, 12.8326}, {2.9884, 12.8326}}	{21.0884, 2.72069}	inf	2177.	2515.94	{2012., 2900.}	21.6205	0.0892114	newly	1116.	1258.81	{935., 1542.}	20.1671	0.0817575	Reff	0.942263	0.796259	{0.421962, 1.38512}	43.339	ar	8796.	9404.84	{9361., 9451.}	6.92177
18	R0	{2.96603, 16.7543}	{2.96102, 15.3328}	{{2.95376, 15.278}, {2.99911, 15.6207}}	{0.525942, 8.4842}	inf	2830.	2481.97	{1828., 2987.}	14.6073	0.0775281	newly	1119.	1066.55	{823., 1307.}	14.2206	0.0669985	Reff	0.741508	0.829558	{0.430067, 1.21311}	43.1559	ar	9394.	9382.76	{9340., 9426.}	0.341501
19	R0	{2.25216, 9.90465}	{2.99939, 13.7076}	{{2.99939, 13.7076}, {2.99939, 13.7076}}	{33.1784, 38.3956}	inf	1991.	2689.05	{2338., 3048.}	41.7192	0.169639	newly	1350.	1308.65	{1069., 1529.}	12.2748	0.070293	Reff	0.932845	1.00591	{0.547389, 1.70905}	36.985	ar	8631.	9410.45	{9366., 9453.}	9.03084
20	R0	{2.70837, 18.1923}	{2.7499, 15.3124}	{{2.7499, 15.3124}, {2.7499, 15.3124}}	{1.53339, 15.8303}	inf	2671.	2288.1	{1773., 2665.}	14.5662	0.0791199	newly	986.	1005.15	{769., 1207.}	16.0587	0.0723039	Reff	0.903241	0.937499	{0.492782, 1.57954}	39.7674	ar	9183.	9203.37	{9154., 9249.}	0.398527
21	R0	{2.30288, 15.5877}	{2.85956, 19.8999}	{{2.85956, 19.8999}, {2.85956, 19.8999}}	{24.1732, 27.6641}	inf	2049.	2219.85	{1615., 2782.}	19.6753	0.0857373	newly	836.	740.148	{535., 977.}	19.0466	0.0960644	Reff	0.842854	0.72773	{0.402912, 1.12638}	43.1677	ar	8674.	9302.69	{9253., 9345.}	7.24797
22	R0	{2.82208, 10.7544}	{2.81311, 10.4354}	{{2.81311, 10.4354}, {2.81311, 10.4354}}	{0.317851, 2.96623}	inf	2783.	2226.52	{1592., 2653.}	19.9955	0.106728	newly	1622.	1343.26	{938., 1629.}	19.1739	0.0985159	Reff	0.73233	0.770871	{0.442221, 1.34354}	40.5529	ar	9333.	9267.67	{9208., 9332.}	0.744582
23	R0	{1.69923, 10.1366}	{2.91349, 17.8379}	{{2.77502, 16.515}, {2.98125, 18.4749}}	{71.4596, 75.9756}	inf	1080.	2166.44	{1613., 2816.}	102.058	0.29722	newly	667.	793.571	{582., 1054.}	27.1816	0.103777	Reff	0.906029	0.667297	{0.365545, 1.17205}	48.5287	ar	7055.	9337.11	{9221., 9427.}	32.3474
24	R0	{2.64944, 19.054}	{2.86656, 18.9813}	{{2.86656, 18.9813}, {2.86656, 18.9813}}	{8.19494, 0.381547}	inf	2322.	2010.41	{1453., 2625.}	19.5938	0.100031	newly	778.	691.426	{480., 953.}	21.4569	0.10661	Reff	0.697333	0.615949	{0.348287, 1.20052}	45.992	ar	9147.	9306.52	{9263., 9354.}	1.74401
25	R0	{2.53191, 10.9214}	{2.8833, 17.1847}	{{2.8833, 17.1847}, {2.8833, 17.1847}}	{13.8785, 57.3489}	inf	2411.	2452.79	{1814., 2851.}	17.6248	0.0973663	newly	1327.	979.754	{737., 1153.}	26.1677	0.150107	Reff	0.740077	1.09858	{0.563108, 1.95545}	58.5762	ar	9009.	9313.56	{9263., 9361.}	3.38059
26	R0	{1.89155, 6.76944}	{2.95705, 12.2473}	{{2.91946, 11.8546}, {2.99519, 12.6436}}	{56.3296, 80.9205}	inf	989.	2261.61	{1577., 2863.}	134.239	0.376086	newly	913.	1170.85	{753., 1622.}	39.0512	0.153128	Reff	0.863871	0.744574	{0.348041, 1.32047}	47.7533	ar	7348.	9373.85	{9310., 9429.}	27.5701
27	R0	{2.63149, 15.6979}	{2.96947, 15.963}	{{2.96947, 15.963}, {2.96947, 15.963}}	{12.8437, 1.68876}	inf	2448.	2512.32	{1970., 2939.}	16.7115	0.0910019	newly	1086.	1054.13	{778., 1265.}	14.8072	0.0787632	Reff	0.945758	0.963468	{0.502434, 1.73536}	43.9573	ar	9104.	9383.39	{9320., 9450.}	3.06884
28	R0	{2.85141, 12.6992}	{2.92209, 10.5085}	{{2.91001, 10.3908}, {2.93906, 10.5991}}	{2.50557, 17.2504}	inf	2712.	1690.31	{960., 2556.}	37.9983	0.230453	newly	1362.	946.191	{499., 1581.}	36.4851	0.213673	Reff	0.727965	0.441307	{0.272086, 0.678041}	49.3529	ar	9317.	9353.51	{9291., 9405.}	0.50619
29	R0	{2.54244, 12.9085}	{2.93488, 18.8445}	{{2.93488, 18.8445}, {2.93488, 18.8445}}	{15.4356, 45.9852}	inf	2318.	2486.97	{1227., 2937.}	24.6206	0.129169	newly	1154.	908.656	{626., 1085.}	21.2603	0.127863	Reff	0.769342	1.12714	{0.559095, 2.33558}	58.2135	ar	9034.	9360.51	{9324., 9401.}	3.61422
30	R0	{2.10982, 11.7636}	{2.93488, 18.8445}	{{2.93488, 18.8445}, {2.93488, 18.8445}}	{39.1057, 60.1933}	inf	1626.	2388.89	{1786., 2868.}	53.7132	0.18916	newly	882.	848.18	{593., 1039.}	15.7894	0.0763599	Reff	0.892032	0.889245	{0.437297, 1.96079}	45.8738	ar	8224.	9360.51	{9324., 9401.}	13.8194
31	R0	{2.76312, 15.5396}	{2.99939, 13.7076}	{{2.99939, 13.7076}, {2.99939, 13.7076}}	{8.55084, 11.7892}	inf	2551.	2420.37	{1878., 2880.}	13.6879	0.0729483	newly	1066.	1121.89	{816., 1415.}	18.267	0.0808068	Reff	0.809318	0.693981	{0.39442, 1.10018}	41.4713	ar	9220.	9410.45	{9366., 9453.}	2.06564
32	R0	{2.21727, 15.5439}	{2.46477, 18.9032}	{{2.46477, 18.9032}, {2.46477, 18.9032}}	{11.1624, 21.6117}	inf	1810.	1864.48	{1335., 2246.}	19.6657	0.109676	newly	784.	666.1	{443., 824.}	18.0357	0.113725	Reff	0.888682	0.897567	{0.478658, 1.46876}	37.1238	ar	8359.	8883.57	{8780., 8947.}	6.27547
33	R0	{2.72912, 10.8159}	{2.99939, 13.7076}	{{2.99939, 13.7076}, {2.99939, 13.7076}}	{9.90319, 26.7356}	inf	2166.	2420.37	{1878., 2880.}	20.5262	0.0911281	newly	1179.	1121.89	{816., 1415.}	16.3411	0.0798616	Reff	0.537091	0.693981	{0.39442, 1.10018}	44.3879	ar	9196.	9410.45	{9366., 9453.}	2.33201
34	R0	{2.20928, 10.1308}	{2.93906, 10.3908}	{{2.93906, 10.3908}, {2.93906, 10.3908}}	{33.0325, 2.56643}	inf	1753.	1533.2	{992., 2441.}	28.2944	0.13978	newly	1080.	846.016	{514., 1380.}	35.0865	0.185255	Reff	0.754911	0.386356	{0.268336, 0.586636}	53.4423	ar	8388.	9363.79	{9316., 9404.}	11.6331
35	R0	{2.10103, 15.2673}	{2.8833, 17.1847}	{{2.8833, 17.1847}, {2.8833, 17.1847}}	{37.2327, 12.5589}	inf	1737.	1798.7	{1165., 2523.}	22.4714	0.0970123	newly	782.	669.016	{420., 1007.}	26.3427	0.13421	Reff	0.894198	0.511776	{0.308513, 0.806171}	54.0718	ar	8264.	9313.56	{9263., 9361.}	12.7004
36	R0	{2.74914, 19.2775}	{2.86087, 19.9873}	{{2.86095, 19.9929}, {2.86095, 19.9929}}	{4.06406, 3.68212}	inf	2701.	2235.6	{1584., 2808.}	18.7128	0.101387	newly	946.	739.567	{536., 971.}	23.0691	0.124219	Reff	0.859106	0.719391	{0.384226, 1.00448}	42.4415	ar	9301.	9304.53	{9252., 9356.}	0.343332
37	R0	{2.78161, 16.0136}	{2.85895, 15.567}	{{2.86118, 15.5722}, {2.86118, 15.5722}}	{2.82616, 2.78885}	inf	2648.	2325.59	{1806., 2728.}	13.5524	0.0698782	newly	1097.	976.361	{770., 1183.}	14.4253	0.0714538	Reff	0.778294	0.784679	{0.447775, 1.44404}	39.8184	ar	9240.	9308.	{9255., 9350.}	0.744447
38	R0	{2.4454, 8.33501}	{2.9884, 12.8326}	{{2.9884, 12.8326}, {2.9884, 12.8326}}	{22.205, 53.9602}	inf	1846.	2515.94	{2012., 2900.}	39.1203	0.143949	newly	1286.	1258.81	{935., 1542.}	13.8865	0.0645797	Reff	0.652922	0.796259	{0.421962, 1.38512}	43.6943	ar	8853.	9404.84	{9361., 9451.}	6.23335
39	R0	{2.9154, 12.3861}	{2.91946, 11.8546}	{{2.91946, 11.8546}, {2.91946, 11.8546}}	{0.13926, 4.2911}	inf	2561.	2249.63	{1693., 2776.}	16.5144	0.0820405	newly	1272.	1185.57	{890., 1546.}	18.1368	0.085244	Reff	0.603196	0.670765	{0.378654, 1.00371}	41.164	ar	9371.	9349.67	{9293., 9392.}	0.360687
40	R0	{2.40923, 15.1963}	{2.86118, 15.5722}	{{2.86118, 15.5722}, {2.86118, 15.5722}}	{18.7591, 2.47363}	inf	2019.	2333.48	{1806., 2728.}	22.0845	0.0891807	newly	887.	980.	{770., 1183.}	17.8775	0.0728281	Reff	0.893824	0.786257	{0.447775, 1.44404}	42.2654	ar	8735.	9309.66	{9255., 9350.}	6.57877
41	R0	{2.63137, 15.238}	{2.8833, 17.1847}	{{2.8833, 17.1847}, {2.8833, 17.1847}}	{9.5741, 12.7753}	inf	2300.	2112.21	{1456., 2696.}	16.7149	0.0830016	newly	964.	799.869	{558., 1086.}	21.9475	0.114536	Reff	0.798621	0.652009	{0.361566, 1.1242}	45.9079	ar	9150.	9313.56	{9263., 9361.}	1.78751
42	R0	{2.48769, 19.4593}	{2.64373, 19.7855}	{{2.64373, 19.7855}, {2.64373, 19.7855}}	{6.27249, 1.67632}	inf	2205.	1985.97	{1488., 2441.}	15.832	0.0782071	newly	727.	670.54	{491., 856.}	16.9188	0.0812408	Reff	0.77392	0.761675	{0.440181, 1.28538}	40.4498	ar	8932.	9088.49	{9032., 9142.}	1.75204
43	R0	{2.61199, 10.214}	{2.81311, 10.4354}	{{2.81311, 10.4354}, {2.81311, 10.4354}}	{7.69988, 2.16761}	inf	2530.	2226.52	{1592., 2653.}	14.631	0.0764753	newly	1603.	1343.26	{938., 1629.}	18.5615	0.094891	Reff	0.846024	0.770871	{0.442221, 1.34354}	42.9661	ar	9061.	9267.67	{9208., 9332.}	2.2809
44	R0	{2.50994, 18.3382}	{2.64373, 19.7855}	{{2.64373, 19.7855}, {2.64373, 19.7855}}	{5.33041, 7.89227}	inf	2193.	2114.49	{1722., 2533.}	13.4309	0.0673066	newly	810.	727.54	{567., 887.}	15.1969	0.0766803	Reff	0.937463	0.920039	{0.51024, 1.59576}	39.9659	ar	8827.	9088.49	{9032., 9142.}	2.96241
45	R0	{2.44283, 16.8338}	{2.8833, 17.1847}	{{2.8833, 17.1847}, {2.8833, 17.1847}}	{18.0311, 2.0845}	inf	2140.	2360.36	{1766., 2824.}	21.2747	0.0943128	newly	873.	909.639	{694., 1136.}	17.507	0.0783651	Reff	0.849616	0.842737	{0.440568, 1.527}	41.8972	ar	8878.	9313.56	{9263., 9361.}	4.90603
46	R0	{2.51815, 18.1462}	{2.85963, 19.9045}	{{2.85956, 19.8999}, {2.85956, 19.8999}}	{13.5607, 9.68991}	inf	2349.	1154.33	{642., 1704.}	52.1229	0.351256	newly	861.	366.267	{204., 538.}	58.1727	0.417592	Reff	0.909556	0.350563	{0.245064, 0.406915}	63.944	ar	8978.	9300.92	{9251., 9343.}	3.59676
47	R0	{2.88885, 16.8987}	{2.93488, 18.8445}	{{2.93488, 18.8445}, {2.93488, 18.8445}}	{1.59337, 11.5145}	inf	2783.	2486.97	{1227., 2937.}	14.1809	0.098283	newly	1104.	908.656	{626., 1085.}	17.7299	0.10878	Reff	0.890344	1.12714	{0.559095, 2.33558}	47.2946	ar	9337.	9360.51	{9324., 9401.}	0.352555
48	R0	{2.58709, 13.8786}	{2.86001, 13.5542}	{{2.86001, 13.5542}, {2.86001, 13.5542}}	{10.5493, 2.33741}	inf	2523.	2377.38	{1848., 2787.}	13.4468	0.0738032	newly	1180.	1162.36	{860., 1428.}	14.6568	0.0698966	Reff	0.854775	0.925035	{0.458174, 1.79866}	44.5346	ar	9146.	9309.77	{9263., 9353.}	1.79062
49	R0	{2.42959, 10.7132}	{2.8186, 12.0293}	{{2.8186, 12.0293}, {2.8186, 12.0293}}	{16.0113, 12.2848}	inf	2209.	2326.03	{1647., 2790.}	18.1515	0.0845599	newly	1386.	1255.13	{902., 1531.}	15.1876	0.0772382	Reff	0.872223	0.866015	{0.436883, 1.59138}	39.9478	ar	8863.	9275.2	{9219., 9322.}	4.6508
50	R0	{2.60513, 13.2796}	{2.86118, 15.5722}	{{2.86118, 15.5722}, {2.86118, 15.5722}}	{9.82868, 17.2641}	inf	2476.	2488.44	{1819., 2859.}	15.4341	0.0818925	newly	1231.	1091.05	{863., 1273.}	13.0921	0.0714752	Reff	0.977184	1.05409	{0.5874, 1.92529}	35.6861	ar	9095.	9309.66	{9255., 9350.}	2.36015
